# Optimization of Human Peripheral Blood Mononuclear Cells (PBMCs) Cryopreservation

**Published:** 2012

**Authors:** Robab Nazarpour, Ebrahim Zabihi, Ebrahim Alijanpour, Zeinab Abedian, Hamid Mehdizadeh, Fatemeh Rahimi

**Affiliations:** 1 *Cellular and Molecular Biology Research Center, Babol University of Medical Sciences, Babol, Iran.*; 2 *Department of Anesthesiology, Roohani Hospital, Babol University of Medical Sciences, Babol, Iran.*; 3 *Department of Pharmacology & Physiology, School of Medicine, Babol University of Medical Sciences, Babol, Iran.*; 4 *Student Research Committee, Babol university of medical Sciences, Babol, Iran.*

**Keywords:** PBMCs, ficol, freezing, DMSO, FBS

## Abstract

Cryopreservation is the method of choice for long term storage of human PBMCs. This study was designed to compare the different combinations of variables affecting the cryopreservation of PBMCs samples.

The viability of PBMCs separated from 2×5 ml peripheral blood samples obtained from 16 healthy adult volunteers, were measured using trypan blue dye exclusion method just before freezing with different concentrations of DMSO (10, 15, and 20%) and FBS (40 or 70%) at two different temperatures (either 4^o^C or 25^o^C). Then after 2 weeks the cells were thawed and the viability was measured again. Also the PBMCs response to PHA was measured after 48 h using MTT assay. The effects of the different variables were calculated and compared among the groups.

A total of 192 PBMCs cryotubes made from blood samples of 16 volunteers were tested. The viability of the cells obtained by the two centrifugation procedure was the same (both more than 99%). The concentration of the FBS (40 vs 70%) did not show to have significant effects on either cells viability or response to PHA. On the other hand 20% DMSO concentration and freezing temperature at 25^o^C decreased both cells.

Based on the obtained results, it is recommended to centrifuge the PBMCs under higher revolt speed at shorter time (700 g for 20 minutes) and decrease the FBS concentration to 40%. The DMSO concentration should be kept at 10-15% and the freezing medium be cooled down to 4^o^C.

Cryopreservation is a branch of cryobiology which uses extremely low temperatures to preserve different types of cells for long term (1-2). This is the only procedure currently available for long term preservation of human and animal cells which is commonly used in different medical areas like: fertility, stem cells research, cord blood and peripheral blood banks (3-6). The freezing mechanism is based on intracellular-ice formation by the reduction temperature of water in the cells without any cryogenic damages. This could effectively stop cell movement and shut down the cell biochemical processes resulting in an increase in the cell survival (2). Optimal freezing procedure for maximum viable cell recovery on thawing depends on: 1- Preventing the intracellular ice crystal formation by slowing the cooling rate (but not so slow to encourage extracellular ice crystals growth), 2- Using a cryoprotectant (e.g. DMSO or glycerol), 3- Storing the cell at lowest temperature (firstly at -80ºC then in liquid nitrogen). The cooling rate (usually 1ºC per minute is recommended), type and concentration of cryoprotectant have the major effects on quality of cryopreservation (2). Generally, freezing medium consists of basal medium supplemented with higher concentrations of serum, i.e. fetal bovine serum (FBS) or bovine serum albumin (BSA), and DMSO as anti-freeze agent.

The storage of peripheral blood mononuclear cells (PBMCs) using the freezing technique is a common laboratory procedure for preserving these cells for phenotypic and functional analysis for a wide range of infectious diseases and clinical vaccine investigations. Various studies have proven that the quality of frozen PBMCs has a vital function on their survival and appropriate freezing technique is the key to the success of the assays using these cells (7).

The purpose of this study was to compare and select the best freezing condition of PBMCs which could preserve the cells with higher viability and biological activity.

## Materials and Methods

**Isolation of Peripheral Blood Mononuclear Cells (PBMCs)**

Peripheral blood samples (10 ml each) of 16 healthy middle aged volunteers who had already given written consent, were transferred aseptically into 50 ml polystyren centrifuge tubes containing EDTA (EthyleneDiamineTetra-Acetic Acid; Sigma- Aldrich, UK) as anticoagulant and gently mixed. In a laminar air flow cabinet, same volume (10 ml) of D-PBS solution was added into the tubes and 5ml of this diluted blood samples were gently layered and isolated by density centrifugation on Ficoll-Hypaque® gradients (Sigma- Aldrich, UK) using two different centrifugation programs at room temperature (20 minutes at 700 g or 30 minutes at 400 g). The PBMCs layer was collected and washed by PBS (250 g for 10 min) then resuspended in 2ml RPMI-1640 medium (PAA, Austria) and the cell viability was determined soon after using trypan blue dye exclusion and PBS as the diluent (8).

**PBMCs Cryopreservation**

After preparing PBMCs suspension in RPMI-1640 (2×10^6 ^cells/ml), 12 different freezing conditions were used for each blood sample PBMCs based on: the temperature of freezing medium (4 or 25ºC), the concentration of FBS (40 or 70%) and the concentration of DMSO (10 or 15 or 20%) according to table 1. 

**Thawing the PBMCs and secondary viability assay**

After two weeks cryopreservation in liquid nitrogen, the PBMCs were thawed in a 37˚C water bath, and the cells were diluted in pre-warmed complete medium (RPMI-1640+10% FBS). Then the cell viability was measured using Trypan blue dye exclusion technique.

**PBMCs phytohaemagglutinin (PHA) stimulation assay**

The thawed cell suspension was washed by 6 times volume complete medium (250 g for 10 min). Then the cells were seeded in a 24 wells plate (6×10^5^ cells/well) in 1 ml complete medium with 10 µg/ml PHA. After 48 h incubation, 200 µl of MTT [3-(4,5- Dimethylthiazol -2-yl) -2,5-Diphenyltetrazoli-um) solution (5 mg/ml) in PBS was added to each well and after 3.5 h incubation at 37˚C, gently 1.1 ml of the supernatant was removed and 800 µl of acidic isopropanol (0.04 N, HCl) was used to dissolve the formazan precipitate and the resulting purple color absorbance was red at 570 and 630 nm (the second wavelength was used for eliminating the nonspecific absorbance) using a spectrophotometer (Camspec®, UK).

**Table 1:** Twelve freezing conditions (freezing medium temperature, DMSO and FBS concentration) used for cryopreservation of PBMCs for each sample

Group Number	Group code	FBS (%)	DMSO (%)	Freezing Medium temp. (ºC)
I	401004	40	10	4
II	401025	40	10	25
III	401504	40	15	4
IV	401525	40	15	25
V	402004	40	20	4
VI	402025	40	20	25
VII	701004	70	10	4
VIII	701025	70	10	25
IX	701504	70	15	4
X	701504	70	15	25
XI	702004	70	20	4
XII	702025	70	20	25

After gently mixing the cells corresponding freezing medium for each group, the PBMCs were transferred to 1.5 ml Cryotube® vials (Nalgene, UK) and stored in -80ºC overnight. Then the cryotubes were quickly transferred to a liquid nitrogen Dewar

**Statistical analysis**

One way Analysis of Variances (ANOVA) with Dunnet’s post hoc test and Factorial-ANOVA were used to determine the different variables effects on (and the highest viability among them) the different cryopreserved PBMCs groups. 

## Results

**Isolation of PBMCs by gradient centrifugation protocols**

The viability of PBMCs isolated by both protocols was more than 98% and there was no significant difference among the two time-RCF protocols used for Ficoll gradient centrifugation of PBMCs.

**Trypan blue test results on revived PBMCs**

Detailed results for viability of different cryopreserved PBMCs groups are shown in Table 1. The highest viability observed in groups III and IX (group codes: 40 15 4 and 70 15 4; code numbers indicate the concentration of FBS, DMSO and freezing-medium temperature respectively). The lowest viability results were observed in groups VI and XII (both with 20% DMSO). Among the 4 different tested variables, the concentration of DMSO showed the highest effect on PBMCs survival after cryopreservation which led to a significant decrease in viability by increasing the DMSO concentration to higher than 15%. The freezing medium temperature seems to have mild effects on the viability and the FBS concentration (from 40 to 70%) in freezing medium does not seem to have any significant effect.

**Fig 1:**
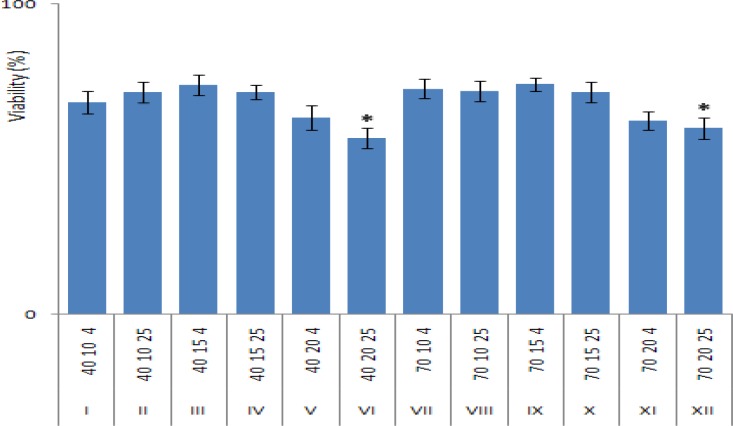
Effects of different cryopreservation conditions on PBMCs viability using trypan-blue dye exclusion. Twelve groups (I to XII) with different concentrations of FBS (40 or 70%), DMSO (10,15, or 20%) at two different freezing medium temperature (4ºC or 25 ºC) have been tested, the code number for each group indicates the variables amount (FBS %-DMSO%-Temperature). Each group contains 16 cryotubes prepared from 16 different blood samples

An asterisk (*) means significant difference (P<0.05) between those groups with highest viability.

**PHA stimulated PBMCs proliferation**

**Fig 2:**
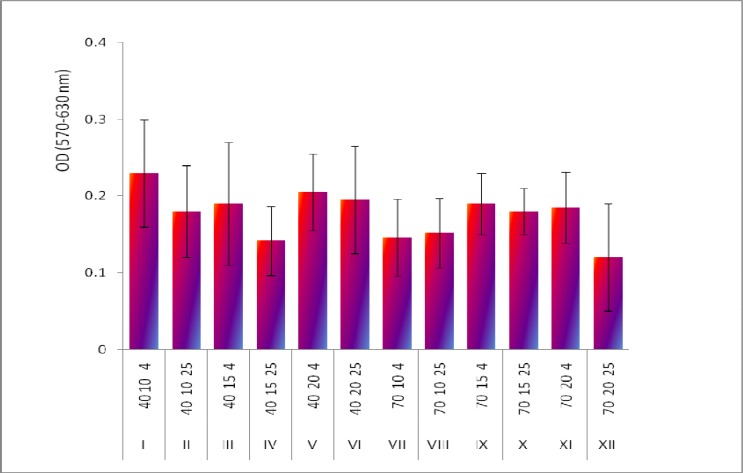
. Effects of different cryopreservation conditions on PBMCs response to phytohaemaglutinan-A (PHA) stimulation measured by MTT assay. Twelve groups (I to XII) with different concentrations of FBS (40 or 70%), DMSO (10,15, or 20%) at two different freezing medium temperature (4ºC or 25 ºC) have been tested, the code number for each group indicates the variables amount (FBS %-DMSO%-Temperature). The MTT assay was performed after 48 h. exposure to PHA. No significant difference was observed between groups

The MTT assay results for different groups have been depicted in Fig.2. No significant differences were observed between each pair groups (P<0.05; using ANOVA with Dunnet’s post hoc test). 

## Discussion & Conclusion

The main factor affecting the viability of cryopreserved PBMCs was found to be the DMSO concentration. The freezing medium temperature could increase the mean of viability but its effect was not significant when studied as a separate main factor. It is now well established that in the mammalian cell banking, the cryoprotective (anti-freezing) agents have essential role for successful cryopreservation. Among the many different cryoprotective chemicals commonly used in cell culture, DMSO is the most common used agent with desirable properties such as hydrophilicity, suitable viscosity, stability, and some other good properties that has made it a universal anti-freezing agent. 

The proposed mechanism for DMSO effectiveness is a reduction of intracellular ice-crystal formation without endangering the cells to water deprivation consequent to freezing (2). However, besides its protective role, DMSO is a toxic substance which could adversely affect the PBMCs viability while its concentration (or its time of exposure to the cells) is increased (2, 6, 9). For this reason, lower than 10% concentrations of DMSO have been tried by many investigators for PBMCs cryopreservation (10-13). However, the minimum concentration of DMSO in the final freezing medium has been suggested to be 10% and lowering the concentration usually adversely affects the cell survival (2). This fact has been proven by comparing 10% DMSO with its lower concentrations as anti-freeze agent which showed better results with at least 10% DMSO concentration (14). On the other hand, increasing the concentration of DMSO up to 20% had a dual trend on cryopreservation quality: while it improved the thermodynamic properties of freezing, its higher concentration might increase the cytotoxicity, hence a decrease in the PBMCs viability. This has been shown by hepatic progenitor cells (15) as well as pancreatic endocrine cells (16).

Based on our findings in this study, while increasing the DMSO concentration up to 15% did not have any adverse effect on PBMCs viability, the higher DMSO concentration (20%) had showed significant harmful effects on the cell survival (Fig.1). 

Since there were no significant differences between 10% and 15% DMSO concentration (as the main factor comparing different groups), we suggest this range of DMSO concentration for PBMCs cryopreservation.

The viability of PBMCs obtained by 2 different centrifugation procedures (either 700g for 20 min or 400 g for 30 min) were both quite high (> 98%) and acceptable. The exposure to toxic Ficoll seems to be negligible during the centrifugation and washing of the cells by PBS at minimum rotational force (250 g for 10 min) soon after gradient centrifugation, can eliminate any Ficoll contamination. 

Oxidative stress remains the most important mechanism of cell damage and consequent decrease in cell viability which is partly related to the type of anticoagulant used during blood sample collection (17). We used EDTA as the anticoagulant agent for all samples which probably induces less oxidative stress compared to the other anticoagulants, i.e. heparin (17). 

The different sources of proteins, often FBS (Fetal Bovin Serum) or BSA (Bovine Serum Albumin) have been added to the freezing medium as a natural protective components in order to increase cell survival with a broad range of concentration from 40 up to 100% (2).

Based on our results, 70% FBS did not show any improvements in the cell viability compared to 40% FBS. Similar results have been reported in previous studies investigating the effects of serum in freezing medium on cell survival which show no positive effect (18-20). On the other hand, in a study conducted on spermatogonial cells freezing medium, an increase in serum concentration from 50 to 70% has increased the cell viability (21).

Another controversy is the freezing medium temperature, while some authors recommend cooling the freezing medium down to 4ºC just before mixing with the cell suspension to decrease the damage induced by toxic DMSO, some others beleive that this cooling does not only have any improving effects on cell survival but also it worsens the cryopreservation stress by preventing the DMSO penetration into the cells (2, 5). In our study, this cooling temperature did not show any significant effect when studied alone but it could have a mild effect when combined with DMSO effects. The positive effects of cold freezing medium on PBMCs functional responses has been already reported (22).

In the current study, the MTT assay results (PHA induced PBMCs proliferation) did not show any significant differences among the groups. This assay basically evaluate the function of PBMCs to proliferate in response to a mitogen (PHA). Since we have used a colorimetric test procedure (formazan formation from MTT salt) the sensitivity of this method may not be high enough to discriminate the minor changes in PBMCs responses. Radio-assays are more reliable and using other methods, e.g. Elispot is recommended for further functional assays on these cells. 

As the final word, the optimum freezing condition of PBMCs has been found out to be 40% FBS and 10-15% DMSO (i.e. 12.5%) with cooling the freezing down to 4ºC. 
